# Early Detection of Cystoid Macular Edema in Retinitis Pigmentosa Using Longitudinal Deep Learning Analysis of OCT Scans

**DOI:** 10.3390/diagnostics16010046

**Published:** 2025-12-23

**Authors:** Farhang Hosseini, Farkhondeh Asadi, Reza Rabiei, Arash Roshanpoor, Hamideh Sabbaghi, Mehrnoosh Eslami, Rayan Ebnali Harari

**Affiliations:** 1 Gastroenterology and Liver Diseases Research Center, Research Institute for Gastroenterology and Liver Diseases, Shahid Beheshti University of Medical Sciences, Tehran 19899-34148, Iran; hosseini.f@sbmu.ac.ir; 2 Department of Health Information Technology and Management, School of Allied Medical Sciences, Shahid Beheshti University of Medical Sciences, Tehran 19899-34148, Iran; 3 Department of Computer, Yadegar-e-Imam Khomeini (RAH) Shahre Rey Branch, Islamic Azad University, Tehran 14778-93855, Iran; arash.roshanpoor@iau.ac.ir; 4 Ophthalmic Epidemiology Research Center, Research Institute for Ophthalmology and Vision Science, Shahid Beheshti University of Medical Sciences, Tehran 19899-34148, Iran; 5 MetaLeaps Inc., Boston, MA 02108, USA; mehrnoosh.eslami@yahoo.com; 6 Quantitative Musculoskeletal Imaging Group Research (Q-MIG), Department Radiology, and Harvard Data Science Initiative, Harvard Medical School, Boston, MA 02115, USA; rharari@bwh.harvard.edu

**Keywords:** retinitis pigmentosa, cystoid macular edema (CME), optical coherence tomography (OCT), deep learning, longitudinal data, early detection

## Abstract

**Background/Objectives**: Retinitis pigmentosa (RP) is a progressive hereditary retinal disorder that frequently leads to vision loss, with cystoid macular edema (CME) occurring in approximately 10–50% of affected patients. Early detection of CME is crucial for timely intervention, yet most existing studies lack longitudinal data capable of capturing subtle disease progression. **Methods**: We propose a deep learning–based framework utilizing longitudinal optical coherence tomography (OCT) imaging for early detection of CME in patients with RP. A total of 2280 longitudinal OCT images were preprocessed using denoising and data augmentation techniques. Multiple pre-trained deep learning architectures were evaluated using a patient-wise data split to ensure robust performance assessment. **Results**: Among the evaluated models, ResNet-34 achieved the best performance, with an accuracy of 98.68%, specificity of 99.45%, and an F1-score of 98.36%. **Conclusions**: These results demonstrate the potential of longitudinal OCT–based artificial intelligence as a reliable, non-invasive screening tool for early CME detection in RP. To the best of our knowledge, this study is the first to leverage longitudinal OCT data for AI-driven CME prediction in this patient population.

## 1. Introduction

Retinitis pigmentosa (RP) is a progressive, inherited retinal dystrophy that primarily affects rod photoreceptors, leading to gradual vision loss [1,2]. While the peripheral vision is typically affected first, the occurrence of cystoid macular edema (CME) can cause additional acute or subacute deterioration in central vision [3]. As a result of fluid buildup in the macula, CME develops, leading to the formation of cyst-like spaces. The exact pathogenesis remains unclear, but proposed mechanisms include macular Müller cell impairment, autoimmune responses, blood-retinal barrier breakdown, vitreous traction, and dysfunction of the retinal pigment epithelial cells (RPE) pump [2,4]. Symptoms typically involve progressive loss of visual field, night vision impairment, and decreased visual acuity due to cone photoreceptor loss, ultimately resulting in central vision deterioration [5,6].

Early detection of CME in RP patients is essential. CME impacts central vision in 25% of cases, worsening vision loss and increasing the need for costly eye care [7,8]. Delayed treatment often requires more invasive procedures with higher risks. Timely screening can prevent CME from becoming chronic, reduce the need for complex treatments, and improve patient outcomes [9,10]. Effective early detection preserves vision, minimizes drug side effects, and enhances treatment prognosis. Failure to diagnose CME promptly may lead to disease progression and resistance to treatment, which requires a more cautious and stepwise approach [2,11]. Early detection of CME in RP patients is crucial and can be effectively achieved with OCT imaging [8]. However, research on CME in RP patients is limited by insufficient RP-specific data and the lack of longitudinal imaging.

OCT offers high-resolution, non-invasive imaging that detects subtle retinal abnormalities like subclinical CME [12]. Spectral-domain OCT (SD-OCT) excels in measuring central foveal thickness and identifying CME without fluorescein dye. Its non-invasive nature, with its ability to monitor retinal changes and treatment response, enhances its utility [11,13]. Additionally, OCT’s safety, speed, and repeatability make it ideal for routine screening and managing CME in RP patients.

Despite these advantages, the complexity of retinal patterns in OCT scans can lead to missed diagnoses during manual review [2,6]. Deep learning (DL), a subset of artificial intelligence (AI), has shown great promise in identifying hidden patterns in OCT images, which can aid in the early detection of CME and improve screening for RP patients [14,15,16,17,18]. The consistent, high-resolution 3D data provided by macular OCTs makes them particularly well-suited for DL applications [19,20]. Furthermore, recent studies have shown the potential of combining DL with telemedicine tools to enhance screening and diagnostic capabilities in primary care settings [21,22,23,24].

Over the past five years, DL for OCT analysis has expanded rapidly, with many studies reporting strong performance in classifying retinal diseases. CNNs have been used to identify DME, CME, AMD, and other macular pathologies from B-scan and volumetric OCT data [25,26]. Recent work includes explainable models such as DeepOCT [27], multitask approaches for DME classification [28], and hybrid CNN-RNN or Transformer models that capture spatial and contextual retinal information [15,29]. Most existing studies focus on cross-sectional diagnosis rather than longitudinal prediction. No prior work has examined future CME development in RP using longitudinal OCT data. This gap motivates the predictive framework proposed in the present study.

Convolutional neural networks (CNNs) are particularly promising, which have shown accuracy rates between 80% and 99% in detecting and segmenting CME in diseases such as diabetic macular edema (DME) and age-related macular degeneration [30]. However, there is a significant research gap in CME in RP patients, despite the high clinical relevance. CME in RP presents unique challenges due to factors such as Müller cell dysfunction and retinal pigment epithelium abnormalities, which differ from other retinal diseases. While early DL models like ResNet and AlexNet have shown promise, their effectiveness is limited by the lack of representative RP data and the progressive [19,31,32], heterogeneous nature of photoreceptor loss in these patients. Most existing studies rely on cross-sectional datasets and overlook the importance of longitudinal imaging, which is vital for identifying subtle, progressive changes that precede the onset of CME. This lack of long-term data hampers early detection and delays timely intervention.

To address this gap, our study leverages a unique longitudinal OCT dataset that includes multiple follow-up visits, enabling the development of predictive DL models tailored to RP. By capturing early patterns and risk factors associated with CME progression, our goal is to support clinicians in making earlier, more informed treatment decisions—ultimately improving patient outcomes.

## 2. Materials and Methods

### 2.1. Data Set

**Figure 1:**
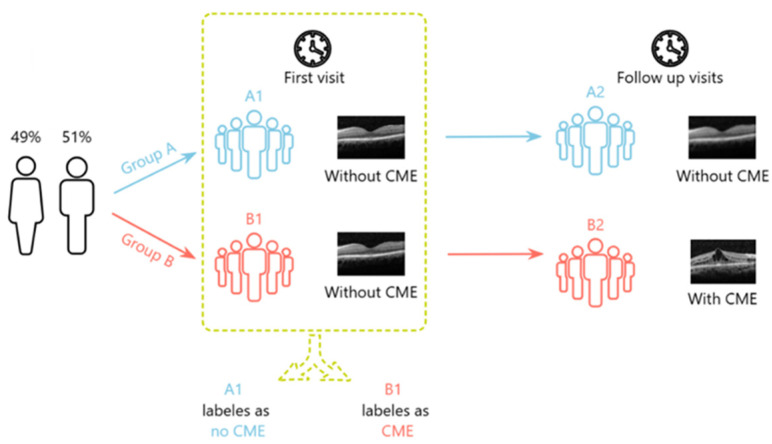
Labeling process for early detection of CME.

In this retrospective cohort study, 570 longitudinal OCT images were collected from 114 eyes diagnosed with RP, with or without CME complications. The study population had a mean age of 42.2 ± 14.43 years and consisted of 51% males. To facilitate the early detection of CME, imaging data from patients’ earlier clinical visits were analyzed and labeled based on the development of CME in follow-up visits. This approach involved reviewing the earlier visit images and categorizing them based on whether CME was subsequently diagnosed. CME was defined as cases in which CME was found in the OCT scans, whereas No CME referred to cases with no CME at the time of imaging. However, No CME does not invariable classify as a normal or healthy retina but includes RP patients with other retinal abnormalities who did not satisfy the clinical criterion for CME. Figure 1 shows the labeling process. Group A includes images from patients without CME at both initial and follow-up visits. Group B includes images from patients without CME initially but with CME in follow-up. This labeling enabled analysis of early OCT images (A1, B1) based on later CME development (A2, B2) to train a DL model for early CME detection in RP patients. Following the guidelines, which required five scans per eye, a total of 270 slides were collected from RP patients with CME complications, alongside 300 slides from patients without CME complications.

The study was approved by the Research Ethics Committees of Vice-Chancellor in Research Affairs—Shahid Beheshti University of Medical Sciences, Tehran, Iran (IR.SBMU.RETECH.REC.1403.608), and informed consent was obtained from all participants. All methods were performed following relevant guidelines and regulations, including the principles outlined in the Declaration of Helsinki. Data were sourced from the Iranian National Registry for Inherited Retinal Diseases (IRDReg^®^) between 2013–2024 [33].

Each OCT scan was independently reviewed and annotated by three retina specialists, each with more than five years of experience. Discrepancies in annotations were resolved through structured consensus meetings. In these meetings the reviewers jointly examined and discussed the cases to arrive at a final agreement on labels. Inter-rater agreement was assessed during a pilot phase to establish consistency across annotators. Additionally, a random subset of annotated scans (10%) was re-evaluated by a senior retina specialist to confirm labeling quality and minimize bias. Identifying information was removed and each patient was assigned a unique number.

Patients were informed about procedures involving pupil dilation, which may temporarily affect vision and were instructed to remain still during imaging for accuracy. Patients with difficulty were given additional support. Operators calibrated imaging devices, positioned patients correctly, and reviewed image quality, repeating the process if necessary. The collected data was divided into two groups: RP patients without CME during follow-up and those who developed CME after the initial consultation. Spectral-domain optical coherence tomography (SD-OCT, Heidelberg Engineering, Heidelberg, Germany) was utilized, employing a 6 × 6 mm 3D macular scan protocol with an image resolution of 400 × 500 pixels.

### 2.2. Data Preparation

We performed image resizing, data augmentation, and noise removal to prepare the dataset for improved model performance. The optimal size for resizing OCT images depends on the DL model, image characteristics, and computational resources. OCT images, being high-resolution, often require resizing to reduce complexity and memory usage [34,35]. Studies suggest common resizing dimensions of 244 × 244 or 256 × 256 pixels, suitable for CNN architectures, including pre-trained models like those on ImageNet [36,37,38]. We extracted the OCT images from the Heidelberg device to focus on the retinal cross-section and then resized them to 224 × 224 pixels for uniform analysis.

**Figure 2:**
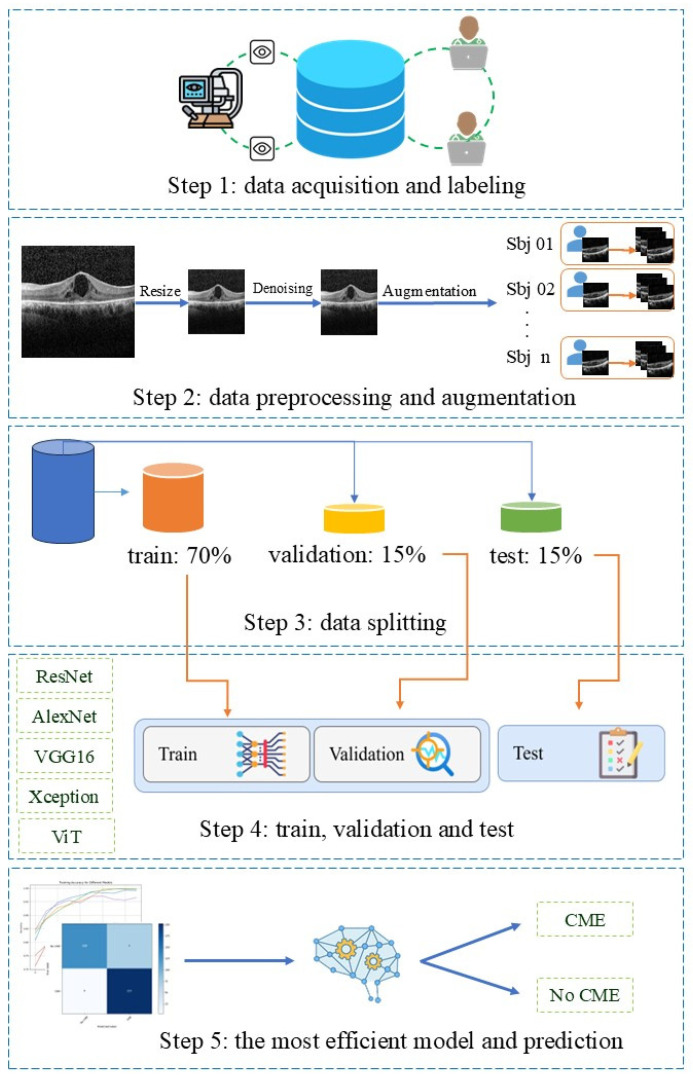
Stages of the proposed methodology.

We use Fast Non-Local Means Denoising (fastNlMeansDenoising) from OpenCV (version 4.10.3) to reduce noise, employing a 7 × 7 window and a 21 × 21 search window. Additionally, we adjust contrast and brightness with alpha = 1.0 and beta = 0 to ensure uniform light intensity (Figure 2, step 2). All OCT images were organized into patient-specific folders before preprocessing to prevent data leakage. Data augmentation (rotation, flipping, and scaling) was applied only within each patient’s folder so that original and augmented images remained grouped by patient. After augmentation, a strict patient-wise split was performed: each patient’s entire folder was assigned exclusively to either the training (70%), validation (15%), or test (15%) subsets. This resulted in 40 patients in the training set, 9 patients in the validation set, and 8 patients in the test set. No images from the same patient appeared in more than one subset. This procedure expanded the dataset, balanced the class distribution, and ensured the complete elimination of both image-level and subject-level leakage. The number of OCT scans per patient was not uniform due to variations in scan quality and availability. Consequently, the test subset contained 8 patients who collectively contributed 68 original OCT scans. After augmentation, this resulted in 342 test images, as reflected in the confusion matrix.

### 2.3. Deep Learning Models

To ensure a representative comparison across classical, residual, efficient, and transformer-based architectures, six widely validated models, including ResNet-18, ResNet-34, VGG16, AlexNet, Xception, and Vision Transformer (ViT), were selected. Their use is supported by prior OCT-based DL research and recent reviews, including our own systematic review on AI for CME detection [19], which identified these architectures as the most frequently applied and best-performing families in retinal OCT analysis. ResNet models offer stable training through residual connections, VGG16 and AlexNet serve as well-established baselines, Xception provides efficient depthwise-separable convolutions suitable for retinal structure modeling, and ViT introduces global self-attention, which has recently shown promise in ophthalmic imaging. A concise overview of the architectural distinctions and motivations for their inclusion is provided in Table 1. These six architectures were implemented and evaluated for their effectiveness in early CME detection in patients with RP [19,39,40]. These models are widely used in transfer learning since they have been pre-trained on large datasets like ImageNet [41]. These models have shown higher performance in ophthalmic image processing. Although ResNet-18, VGG16, and AlexNet are older, they still perform well with OCT images [19,42,43,44]. The dataset was split into training, testing, and validation sets in a 70:15:15 ratio using the sci-kit-learn library, employing a subject-wise splitting method to ensure that images from the same patient were only present in one of the subsets. A fixed random seed was used for all stochastic processes including dataset splitting, weight initialization, and data augmentation to ensure reproducibility. This kept initial conditions identical across architectures, reduced variability, and enabled reliable model comparisons. The models received SD-OCT images of RP patients, both with and without CME complications, as input and generated a numerical output, providing a real value between 0 and 1 for each prediction. All deep learning models were trained using a unified and standardized training setup to ensure a consistent baseline for architectural comparison [19,41]. Input OCT images were resized to 224 × 224 pixels, a dimension widely adopted for CNN architectures transferring from ImageNet [36,38]. The loss function used was nn.CrossEntropyLoss() from the PyTorch (version 2.0.1) library. Training was conducted for a maximum of 30 epochs, employing an early stopping mechanism with a patience of five epochs based on validation loss to prevent overfitting.

Regarding optimization, we evaluated both Adam and SGD, strictly following protocols established in recent OCT classification literature [19,39,40]. Adam was utilized with default parameters (beta_1 = 0.9, beta_2 = 0.999) and a learning rate of 0.001. SGD was configured with a learning rate of 0.01, momentum of 0.9, and weight decay of 1 × 10^−4^. These configurations are consistent with standard practices in medical image analysis [20,34]. A batch size of 32 was chosen to balance GPU memory constraints with convergence stability. While we acknowledge that extensive hyperparameter tuning per model could yield marginal gains, we adopted a fixed-hyperparameter strategy. This approach isolates the impact of the architectural design as the primary variable, minimizing the bias introduced by unequal optimization efforts across different models [20,32].

(Figure 2, Step 3, 4).

**Table 1:** Architectural distinctions and rationale for selecting the six dl models evaluated in this study.

Model	Architectural Characteristics	Rationale for Inclusion
ResNet-18/ResNet-34	Residual blocks, identity skip connections, stable deep training	Most widely used OCT classifiers, robust to overfitting, strong performance in CME/DME tasks
VGG16	Deep sequential 3 × 3 conv blocks, high parameter count	Classical OCT baseline; widely used for transfer learning on OCT
AlexNet	Early CNN with large initial filters, shallow architecture	Lightweight baseline, useful historical benchmark in OCT literature
Xception	Depthwise-separable convolutions, efficient feature extraction	Strong performance with limited OCT data, captures localized retinal patterns efficiently
ViT	Patch embeddings + self-attention, global context modeling	Modern architecture; relevant to OCT where global spatial relations matter, emerging in ophthalmology

### 2.4. Performance Evaluation and Statistical Analysis

Model performance was evaluated on a separate validation set during training and on a test set post-training, as shown in Figure 2 steps 3 and 4. Receiver operating characteristic (ROC) curves were generated to visualize the tradeoff between sensitivity and specificity. Accuracy, specificity, precision, recall, F1-score, and a confusion matrix including True Positive (TP), False Positive (FP), False Negative (FN), and True Negative (TN) were used to assess model efficiency and classification performance.

## 3. Results

### 3.1. This Patient and Imaging Characteristics

The study consists of 57 patients, including 27 with CME and 30 without CME, contributing data from a total of 114 eyes. Each eye was represented by 5 SD-OCT slides, with 270 from eyes with CME and 300 from eyes without CME. Through data augmentation, the total number of images increased to 2280, maintaining a balanced distribution.

### 3.2. Training Loss and Accuracy

**Figure 3:**
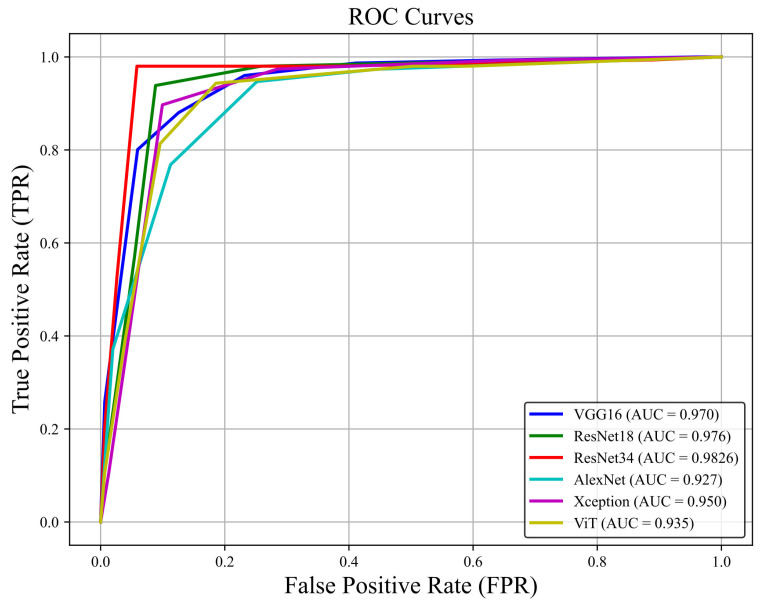
Receiver operating characteristic curves for early detection of CME in RP patients with DL models.

The ROC curves derived from the testing process of models are presented in Figure 3. The average area under the ROC curve (AUC) for all predictors was 0.95. Among the 6 algorithms analyzed, ResNet-34, ResNet-18, and VGG16 achieved the highest AUC indices of 0.98, 0.97, and 0.97, respectively.

**Figure 4:**
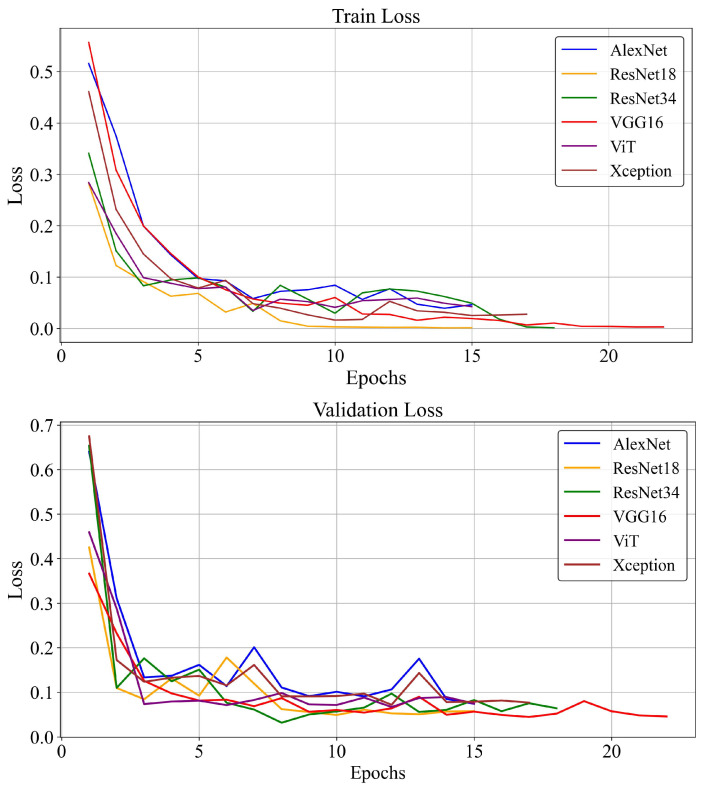
Training and validation loss of different models.

ResNet-34 and ResNet-18 had the lowest loss values, 0.014 and 0.019, respectively, significantly lower than other models, which had a minimum loss of 0.021. Figure 4 illustrates this comparative loss analysis.

**Figure 5:**
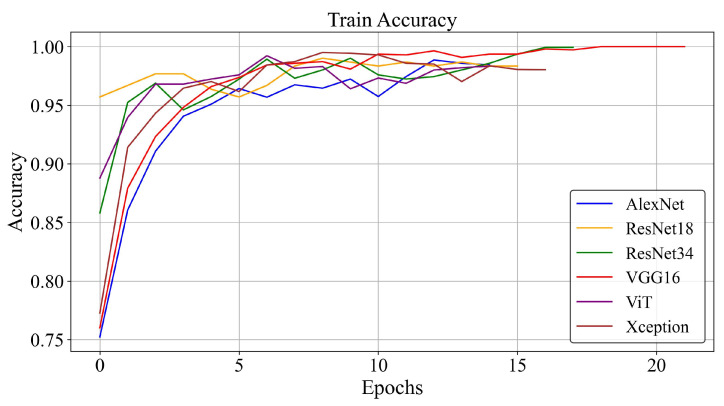

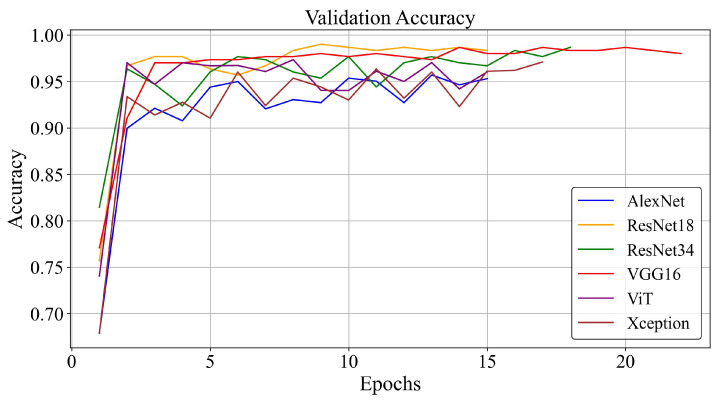
Training and validation accuracy of different models.

Similarly, ResNet-34, ResNet-18, and VGG16 achieved the highest accuracy values (0.986, 0.976, and 0.970). As shown in Figure 5, ViT, Xception, and AlexNet architectures had lower accuracy compared to these models.

### 3.3. Detection Performance

In addition, to check the predictive and detection performance of the models, the indicators of specificity, precision, F1-score, recall, and accuracy based on the best performance of the optimizer were examined, the full details of which are given in Table 2.

Overall, ResNet-34, ResNet-18, and VGG16 provided the best overall accuracy, reaching 0.986, 0.976, and 0.97, respectively. VGG16 achieved high precision (0.9931) and specificity (0.9934), minimizing false positives. Its F1-score (0.9695) and recall (0.947) indicate strong true positive detection but with some missed cases. Accuracy (0.9702) confirms overall reliability. Using the ADAM optimizer, VGG16 is optimal for reducing false positives while maintaining high sensitivity in CME detection. Xception and ViT provided balanced performance, with Xception achieving 0.9603 specificity and 0.9404 recall, and ViT reaching 0.946 recall. AlexNet had moderate accuracy (0.9272) but the lowest recall (0.8742). Overall, ResNet-34 is optimal for minimizing false positives, ResNet-18 is best for detecting true positives, and VGG16, Xception, and ViT offer strong general-purpose performance for CME screening.

The choice of optimizer impacts model performance, with SGD and ADAM being used across different architectures. ResNet-34 and ResNet-18, which achieved the highest overall accuracy (0.9868 and 0.9765, respectively), performed equally well with both SGD and ADAM, indicating their robustness to optimizer selection. VGG16, Xception, and AlexNet, all trained with ADAM, showed strong precision and specificity, with VGG16 achieving the highest precision (0.9931) and specificity (0.9934). ViT, optimized with SGD, had lower precision (0.9259) and accuracy (0.9356) compared to ADAM-optimized models, suggesting that ADAM may provide better overall performance for CME detection. Models trained with ADAM generally showed higher specificity and precision, reducing false positives. The results suggest that while ResNet architectures are optimizer-independent, ADAM tends to enhance performance in models where reducing false positives is critical.

**Figure 6:**
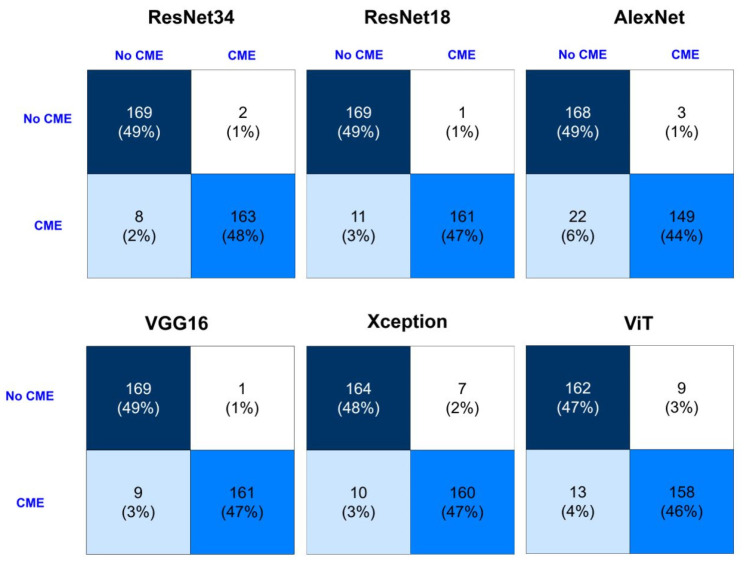
Confusion matrix comparing CME/NoCME labels and model predictions.

The confusion matrices for different models show the classification of cases with and without CME. The best results were achieved by a ResNet-34 (accuracy of 98.68% and F1-score of 98.36%), while the model with a ViT showed relatively low performance at 93.56% accuracy. ResNet-based models and VGG16 maintained high recall and specificity which means equal reduction in both false positive and false negative. However, certain models like AlexNet showed low recall (87.42%) due to difficulties in detecting some CME cases. In general, among different models, evaluation metrics from the confusion matrix result are also well matched, advanced ones like ResNet-34 and VGG16 would be more favorable for a more precise classification of CME (Figure 6).

## 4. Discussion

RP progressively affects rods and cones, with CME contributing to visual impairment in 10–50% of cases [3,45]. While DL models have shown promise in detecting CME in DME and age-related macular degeneration, there is a clear gap in studies focused on CME in RP. Although recent DL studies have reported high performance in OCT-based detection of DME, AMD, and other macular disorders, these works remain predominantly cross-sectional and do not explore longitudinal prediction. This important gap in the literature highlights the relevance and novelty of our RP-specific longitudinal framework. Existing work often relies on cross-sectional data, which fails to capture the dynamic nature of CME progression. Furthermore, many models are trained on single-slice OCT inputs, limiting their ability to detect spatially variable fluid patterns. In contrast, our study leveraged longitudinal OCT data and multi-section inputs, allowing for improved detection of early CME changes across layers such as INL and ONL. Among DL models developed in this study, ResNet showed the highest performance in early detection of CME. These findings address a critical gap in the literature and demonstrate the potential of longitudinal DL approaches in improving CME detection and management in RP. While our results show strong performance for early CME detection in RP, direct numerical comparison with prior OCT studies is not appropriate because those studies involve different diseases, imaging conditions, and datasets. Our reported accuracy reflects performance within the specific context of RP-associated CME, not a head-to-head comparison with other retinal disease studies.

Although our model achieved high accuracy (AUROC 0.98), these results should not be interpreted as superiority over studies on other retinal diseases or populations. Differences in disease mechanisms, imaging devices, dataset size, and class balance prevent direct numerical comparison. Performance values from non-RP datasets are used only as contextual benchmarks.

Strengths of this study include the use of a longitudinal RP dataset, strict patient-wise splitting, and evaluation of multiple established architectures under a unified training pipeline. Limitations include the small RP sample size, lack of external validation, and the rarity of RP-associated CME, which limits generalizability. These points motivate future work involving multicenter datasets, cross-device external validation, longer longitudinal follow-up, and integration of explainable AI tools.

Models based on DL have been explored for diagnosing CME in various retinal diseases, but few studies have focused on rare diseases like RP [19]. DL models require substantial amounts of well-labeled data for effective training and validation to detect subtle patterns. While public datasets such as the 1000 OCT images used by Ahmed et al. for diabetic CME detection [46], provide value, private datasets often offer improved model performance due to their specificity, clinical relevance, and contemporaneity [47,48]. Our study used a unique dataset of 2280 OCT images from RP patients, collected under expert supervision. To the best of our knowledge, it is the first to identify early CME in patients with RP. Additionally, we used five scans from each eye to train models for CME prediction, which differentiates our approach from studies focused on the detection of CME in other retinal diseases.

Compared to previous studies on CME detection in non-RP populations, our model outperformed several benchmarks. For instance, Kaothanthong et al. reported an accuracy of 94.8%, while Bai et al. achieved AUROC of 0.99 in detecting CME across broader retinal conditions [16,39]. Our ResNet-34 model achieved an AUROC of 0.98, sensitivity of 97.12%, and specificity of 99.45%, indicating strong potential for early CME detection in RP patients. The comparative overview of our study with previously published works in this area of AI-assisted retinal disorder detection using retinal OCT images is presented in Table 3. In our study, the ResNet-34 model showed the best performance among all implemented algorithms with values of sensitivity, specificity, and AUROC of 97.12%, 99.45%, and 0.98%.

Table 3 provides a qualitative overview, as the included studies differ in sample size, patient populations, imaging protocols, and disease categories, and therefore incorporate OCT-based works from other retinal diseases for contextual comparison. The 2280 images in the current study represent the fully preprocessed and augmented set derived from 570 original OCT scans.

To develop an effective CME screening strategy for community settings, it is important to think about several key factors. This includes the impact of misdiagnoses, the burden of false positives on specialized care resources, and cost-effectiveness [15]. Among the evaluated models for screening early detection of CME in RP patients, ResNet-34 showed the highest accuracy of 0.98 and specificity of 0.99, making it the most reliable for identifying true negatives that offer strong general-purpose performance for CME screening.

ADAM enhanced precision and specificity in most architectures, which suggests its effectiveness in reducing false positives, particularly in models like VGG16, Xception, and AlexNet. In contrast, ResNet models maintained consistently high accuracy regardless of optimizer choice, indicating their robustness and adaptability. The lower precision and accuracy of ViT with SGD further reinforce ADAM’s advantage in optimizing performance for CME detection. These findings suggest that while ResNet architectures offer stability across optimizers, ADAM is preferable for models where high specificity and precision are critical.

Our study identified CME early in patients with RP. Unlike many DL studies that focus on CME in non-RP populations, particularly diabetic patients, this study offers preliminary evidence that could help monitor patients and their family members at risk for RP complications. Moreover, the longitudinal nature of the dataset used in this study adds a novel dimension to AI-driven CME screening. By incorporating temporal data, our study offers a more comprehensive view of disease progression and highlights the potential of longitudinal follow-up data to improve both sensitivity and specificity in CME detection. Future research should explore how such datasets can be expanded and applied to other rare retinal conditions. One limitation of this study is the absence of external validation datasets. Future research can address this by incorporating data from diverse imaging sources and populations. Although RP with CME complications is rare, we tried to maintain gender balance to ensure fairness and reduce bias. Furthermore, to our knowledge, no public OCT dataset includes RP images with or without CME, and none provide longitudinal follow-up. Available datasets focus on DME, CNV, drusen, or AMD, which differ fundamentally from RP-associated CME in anatomy and pathophysiology. Consequently, external benchmarking with public datasets is not feasible or scientifically relevant for early CME detection in RP. We believe that combining our approach with technologies like augmented reality and telemedicine could significantly improve CME screening for RP patients, both in clinical settings and in remote or underserved areas.

DL models often function as “black boxes,” which makes it difficult to understand how they arrive at their conclusions, especially when detecting CME early in RP patients [49,50]. To improve model interpretability, future studies should use explainable AI (xAI) methods to enhance model transparency [51,52]. By integrating xAI techniques, we may better understand the reasoning behind the DL model’s predictions for CME diagnosis in RP patients. It would be helpful to explore how anatomical data and imaging metrics, such as retinal layer thickness, can aid in predicting CME. Emerging tools like Large Language Models (LLMs) can help integrate imaging data with clinical context to improve model transparency and workflow integration [53]. Additionally, immersive decision support systems using spatial computing and Augmented Reality (AR) may offer a promising path to evaluate and interact with AI-AR fusion tools for patient care in various sets of clinical workflows, such as when multiple caregivers collaborate in diagnosis and treatment processes [54,55,56,57,58]. Finally, studying how these DL models can fit into clinical workflows and affect patient care and outcomes is another vital area for future research [59,60].

## 5. Conclusions

This study investigated how DL models can help in identifying CME at an early stage in patients with RP. Among the various models evaluated, ResNet-34 emerged as the most accurate and specific. These findings highlight the potential for using DL-based OCT analysis in clinical settings to support earlier diagnosis and improve the management of RP-related complications. Future research should aim to evaluate these results by testing more diverse datasets and imaging methods. Using DL-based OCT screening holds potential promise for improving CME detection, particularly in resource-limited environments where early intervention can make a big difference.

## Figures and Tables

**Table 2:** Performance evaluation of DL models in early detection of CME in patients with RP.

Algorithm	Precision	Specificity	F1-Score	Recall	Accuracy	Optimizer
VGG16	0.9931	0.9934	0.9695	0.947	0.9702	ADAM
Xception	0.9595	0.9603	0.9498	0.9404	0.9503	ADAM
ViT	0.9259	0.9242	0.9363	0.946	0.9356	SGD
AlexNet	0.9778	0.9801	0.9231	0.8742	0.9272	ADAM
ResNet-18	0.9821	0.9816	0.9804	0.9718	0.9765	SGD = ADAM
ResNet-34	0.9943	0.9945	0.9836	0.9712	0.9868	SGD = ADAM

**Table 3:** Comparative analysis of recent studies on AI-based ophthalmic disorder detection.

Study	Objective	Dataset	AI Model(s)	Accuracy	Key Findings
Bai et al. (2022) [16]	AI-assisted auto-detection of 15 retinal disorders	878 OCT scans	Deep learning-based AI model	89.10%	AI-assisted OCT achieved high accuracy and was comparable to retina specialists in detecting multiple retinal disorders
Salaheldin et al. (2024) [44]	Automated detection and grading of papilledema from OCT images	OCT images of papilledema cases	SqueezeNet, AlexNet, GoogleNet, ResNet-50, Custom CNN	98.50%	NA novel cascaded model combining multiple architectures for superior detection and grading of papilledema
Saleh et al. (2022) [30]	Multi-class classification of retinal disorders using OCT images	Public OCT dataset	Transfer learning-based platform	98.40%	Achieved high accuracy across multiple retinal conditions
Kaothanthong et al. (2023) [39]	Comparison of DL-based OCT classification/segmentation	14,327 OCT images from macular diseases	RelayNet, Graph-cut technique, and DL classification models	94.80%	High accuracy with DL-based segmentation before classification
current study	Early detection of CME in RP patients	2280 longitudinal OCT images from RP patients	ResNet-18, ResNet-34, Xception, AlexNet, Vision Transformer (ViT), VGG16	98.68%	ResNet models achieved the highest accuracy (98%) and specificity (99%) for early CME detection

## Data Availability

The data that support the findings of this study are available from the Ophthalmic Research Center, Shahid Beheshti University of Medical Sciences, but restrictions apply to the availability of these data, which were used under license for the current study and so are not publicly available. The data are, however, available from the authors upon reasonable request and with the permission of the Ophthalmic Research Center, Shahid Beheshti University of Medical Sciences. Requests for data should be directed to Farkhondeh Asadi (asadifar@sbmu.ac.ir).

## Abbreviations

The following abbreviations are used in this manuscript:

OCT	Optical Coherence Tomography
RP	Retinitis Pigmentosa
CME	Cystoid Macular Edema
SD-OCT	Spectral-Domain Optical Coherence Tomography
DL	Deep Learning
CNN	Convolutional Neural Network
ViT	Vision Transformer
AUROC/AUC	Area Under the Receiver Operating Characteristic Curve
TP	True Positive
FP	False Positive
TN	True Negative
FN	False Negative
IRDReg^®^	Iranian National Registry for Inherited Retinal Diseases
AI	Artificial Intelligence
xAI	Explainable Artificial Intelligence
AR	Augmented Reality

## References

[1] Sayo A., Ueno S., Kominami T., Nishida K., Inooka D., Nakanishi A., Yasuda S., Okado S., Takahashi K., Matsui S. (2017). Longitudinal study of visual field changes determined by Humphrey Field Analyzer 10-2 in patients with Retinitis Pigmentosa. Sci. Rep..

[2] Arrigo A., Aragona E., Perra C., Bianco L., Antropoli A., Saladino A., Berni A., Basile G., Pina A., Bandello F. (2023). Characterizing macular edema in retinitis pigmentosa through a combined structural and microvascular optical coherence tomography investigation. Sci. Rep..

[3] Chen T.C., Lim W.S., Wang V.Y., Ko M.L., Chiu S.I., Huang Y.S., Lai F., Yang C.M., Hu F.R., Jang J.R. (2021). Artificial Intelligence-Assisted Early Detection of Retinitis Pigmentosa—The Most Common Inherited Retinal Degeneration. J. Digit. Imaging.

[4] Arias J.D., Kalaw F.G.P., Alex V., Yassin S.H., Ferreyra H., Walker E., Wagner N.E., Borooah S. (2023). Investigating the associations of macular edema in retinitis pigmentosa. Sci. Rep..

[5] Markomichelakis N.N., Halkiadakis I., Pantelia E., Peponis V., Patelis A., Theodossiadis P., Theodossiadis G. (2004). Patterns of macular edema in patients with uveitis: Qualitative and quantitative assessment using optical coherence tomography. Ophthalmology.

[6] Chen C., Liu X., Peng X. (2022). Management of Cystoid Macular Edema in Retinitis Pigmentosa: A Systematic Review and Meta-Analysis. Front. Med..

[7] Liew G., Strong S., Bradley P., Severn P., Moore A.T., Webster A.R., Mitchell P., Kifley A., Michaelides M. (2019). Prevalence of cystoid macular oedema, epiretinal membrane and cataract in retinitis pigmentosa. Br. J. Ophthalmol..

[8] Oh J.K., Nuzbrokh Y., Lima de Carvalho J.R., Ryu J., Tsang S.H. (2020). Optical coherence tomography in the evaluation of retinitis pigmentosa. Ophthalmic Genet..

[9] Ruff A., Tezel A., Tezel T.H. (2022). Anatomical and functional correlates of cystic macular edema in retinitis pigmentosa. PLoS ONE.

[10] Shimokawa S., Fujiwara K., Murakami Y., Funatsu J., Nakatake S., Yoshida N., Sonoda K.-H., Ikeda Y. (2020). Effect of topical dorzolamide on cystoid macular edema in retinitis pigmentosa. Ophthalmol. Retin..

[11] Kitahata S., Gocho K., Motozawa N., Yokota S., Yamamoto M., Maeda A., Hirami Y., Kurimoto Y., Kadonosono K., Takahashi M. (2024). Evaluation of photoreceptor features in retinitis pigmentosa with cystoid macular edema by using an adaptive optics fundus camera. PLoS ONE.

[12] Kim Y.J., Joe S.G., Lee D.-H., Lee J.Y., Kim J.-G., Yoon Y.H. (2013). Correlations between spectral-domain OCT measurements and visual acuity in cystoid macular edema associated with retinitis pigmentosa. Investig. Ophthalmol. Vis. Sci..

[13] Mousavi N., Monemian M., Ghaderi Daneshmand P., Mirmohammadsadeghi M., Zekri M., Rabbani H. (2023). Cyst identification in retinal optical coherence tomography images using hidden Markov model. Sci. Rep..

[14] Wu Q., Zhang B., Hu Y., Liu B., Cao D., Yang D., Peng Q., Zhong P., Zeng X., Xiao Y. (2021). Detection of morphologic patterns of diabetic macular edema using a deep learning approach based on optical coherence tomography images. Retina.

[15] Rodríguez-Miguel A., Arruabarrena C., Allendes G., Olivera M., Zarranz-Ventura J., Teus M.A. (2024). Hybrid deep learning models for the screening of Diabetic Macular Edema in optical coherence tomography volumes. Sci. Rep..

[16] Bai J., Wan Z., Li P., Chen L., Wang J., Fan Y., Chen X., Peng Q., Gao P. (2022). Accuracy and feasibility with AI-assisted OCT in retinal disorder community screening. Front. Cell Dev. Biol..

[17] Rouhollahi A., Homaei A., Sahu A., Harari R.E., Nezami F.R. (2025). Ragnosis: Retrieval-augmented generation for enhanced medical decision making. medRxiv.

[18] Harari R., Rajabzadeh-Oghaz H., Hosseini F., Shali M.G., Altaweel A., Haouchine N., Rikhtegar F. (2025). CMANet: Cross-Modal Attention Network for 3-D Knee MRI and Report-Guided Osteoarthritis Assessment. medRxiv.

[19] Hosseini F., Asadi F., Rabiei R., Kiani F., Harari R.E. (2024). Applications of artificial intelligence in diagnosis of uncommon cystoid macular edema using optical coherence tomography imaging: A systematic review. Surv. Ophthalmol..

[20] Viedma I.A., Alonso-Caneiro D., Read S.A., Collins M.J. (2022). Deep learning in retinal optical coherence tomography (OCT): A comprehensive survey. Neurocomputing.

[21] Saleh G.A., Batouty N.M., Haggag S., Elnakib A., Khalifa F., Taher F., Mohamed M.A., Farag R., Sandhu H., Sewelam A. (2022). The role of medical image modalities and AI in the early detection, diagnosis and grading of retinal diseases: A survey. Bioengineering.

[22] Parmar U.P.S., Surico P.L., Singh R.B., Romano F., Salati C., Spadea L., Musa M., Gagliano C., Mori T., Zeppieri M. (2024). Artificial intelligence (AI) for early diagnosis of retinal diseases. Medicina.

[23] Salari F., Rafizadeh S.M., Fakhredin H., Rajabi M.T., Yaseri M., Hosseini F., Fekrazad R., Salari B. (2024). Prediction of substantial closed-globe injuries in orbital wall fractures. Int. Ophthalmol..

[24] Liu X., Zhao C., Wang L., Wang G., Lv B., Lv C., Xie G., Wang F. (2022). Evaluation of an OCT-AI-Based Telemedicine Platform for Retinal Disease Screening and Referral in a Primary Care Setting. Transl. Vis. Sci. Technol..

[25] Manikandan S., Raman R., Rajalakshmi R., Tamilselvi S., Surya R.J. (2023). Deep learning–based detection of diabetic macular edema using optical coherence tomography and fundus images: A meta-analysis. Indian J. Ophthalmol..

[26] Leandro I., Lorenzo B., Aleksandar M., Dario M., Rosa G., Agostino A., Daniele T. (2023). OCT-based deep-learning models for the identification of retinal key signs. Sci. Rep..

[27] Altan G. (2022). DeepOCT: An explainable deep learning architecture to analyze macular edema on OCT images. Eng. Sci. Technol. Int. J..

[28] Tang F., Wang X., Ran A.-r., Chan C.K., Ho M., Yip W., Young A.L., Lok J., Szeto S., Chan J. (2021). A multitask deep-learning system to classify diabetic macular edema for different optical coherence tomography devices: A multicenter analysis. Diabetes Care.

[29] Lee Y.J., Choe S., Wy S., Jang M., Jeoung J.W., Choi H.J., Park K.H., Sun S., Kim Y.K. (2022). Demographics Prediction and Heatmap Generation From OCT Images of Anterior Segment of the Eye: A Vision Transformer Model Study. Transl. Vis. Sci. Technol..

[30] Saleh N., Abdel Wahed M., Salaheldin A.M. (2022). Transfer learning-based platform for detecting multi-classification retinal disorders using optical coherence tomography images. Int. J. Imaging Syst. Technol..

[31] Gan F., Wu F.P., Zhong Y.L. (2023). Artificial intelligence method based on multi-feature fusion for automatic macular edema (ME) classification on spectral-domain optical coherence tomography (SD-OCT) images. Front. Neurosci..

[32] Xu W., Fu Y.-L., Zhu D. (2023). ResNet and its application to medical image processing: Research progress and challenges. Comput. Methods Programs Biomed..

[33] Sabbaghi H., Daftarian N., Suri F., Mirrahimi M., Madani S., Sheikhtaheri A., Khorrami F., Saviz P., Nejad M.Z., Tivay A. (2020). The first inherited retinal disease registry in Iran: Research protocol and results of a pilot study. Int. J. Infect. Dis..

[34] Badar M., Haris M., Fatima A. (2020). Application of deep learning for retinal image analysis: A review. Comput. Sci. Rev..

[35] Lalithadevi B., Krishnaveni S. (2022). Detection of diabetic retinopathy and related retinal disorders using fundus images based on deep learning and image processing techniques: A comprehensive review. Concurr. Comput. Pract. Exp..

[36] Feng D., Chen X., Zhou Z., Liu H., Wang Y., Bai L., Zhang S., Mou X. A preliminary study of predicting effectiveness of anti-VEGF injection using OCT images based on deep learning. In Proceedings of 2020 42nd Annual International Conference of the IEEE Engineering In Medicine & Biology Society (EMBC).

[37] Sotoudeh-Paima S., Jodeiri A., Hajizadeh F., Soltanian-Zadeh H. (2022). Multi-scale convolutional neural network for automated AMD classification using retinal OCT images. Comput. Biol. Med..

[38] Berthet A., Dugelay J.-L. A review of data preprocessing modules in digital image forensics methods using deep learning. In Proceedings of 2020 IEEE International Conference on Visual Communications and Image Processing (VCIP).

[39] Kaothanthong N., Limwattanayingyong J., Silpa-Archa S., Tadarati M., Amphornphruet A., Singhanetr P., Lalitwongsa P., Chantangphol P., Amornpetchsathaporn A., Chainakul M. (2023). The Classification of Common Macular Diseases Using Deep Learning on Optical Coherence Tomography Images with and without Prior Automated Segmentation. Diagnostics.

[40] Bai F., Marques M.J., Gibson S.J. (2017). Cystoid macular edema segmentation of optical coherence tomography images using fully convolutional neural networks and fully connected CRFs. arXiv.

[41] Mukhlif A.A., Al-Khateeb B., Mohammed M.A. (2022). An extensive review of state-of-the-art transfer learning techniques used in medical imaging: Open issues and challenges. J. Intell. Syst..

[42] Kırık F., Demirkıran B., Aslanoğlu C.E., Koytak A., Özdemir H. (2023). Detection and Classification of Diabetic Macular Edema with a Desktop-Based Code-Free Machine Learning Tool. Turk. J. Ophthalmol..

[43] Lin C.-L., Wu K.-C. (2023). Development of revised ResNet-50 for diabetic retinopathy detection. BMC Bioinform..

[44] Salaheldin A.M., Abdel Wahed M., Talaat M., Saleh N. (2024). Deep learning-based automated detection and grading of papilledema from OCT images: A promising approach for improved clinical diagnosis and management. Int. J. Imaging Syst. Technol..

[45] Rim T.H., Lee A.Y., Ting D.S., Teo K.Y.C., Yang H.S., Kim H., Lee G., Teo Z.L., Teo Wei Jun A., Takahashi K. (2022). Computer-aided detection and abnormality score for the outer retinal layer in optical coherence tomography. Br. J. Ophthalmol..

[46] Ahmed Z., Panhwar S.Q., Baqai A., Umrani F.A., Ahmed M., Khan A. (2022). Deep learning based automated detection of intraretinal cystoid fluid. Int. J. Imaging Syst. Technol..

[47] Khan S.M., Liu X., Nath S., Korot E., Faes L., Wagner S.K., Keane P.A., Sebire N.J., Burton M.J., Denniston A.K. (2021). A global review of publicly available datasets for ophthalmological imaging: Barriers to access, usability, and generalisability. Lancet Digit. Health.

[48] Le Quy T., Roy A., Iosifidis V., Zhang W., Ntoutsi E. (2022). A survey on datasets for fairness-aware machine learning. Wiley Interdiscip. Rev. Data Min. Knowl. Discov..

[49] Hosseini F., Asadi F., Emami H., Ebnali M. (2023). Machine learning applications for early detection of esophageal cancer: A systematic review. BMC Med. Inform. Decis. Mak..

[50] Hassija V., Chamola V., Mahapatra A., Singal A., Goel D., Huang K., Scardapane S., Spinelli I., Mahmud M., Hussain A. (2024). Interpreting black-box models: A review on explainable artificial intelligence. Cogn. Comput..

[51] Harari R., Al-Taweel A., Ahram T., Shokoohi H. Explainable AI and Augmented Reality in Transesophageal Echocardiography (TEE) Imaging. In Proceedings of 2024 IEEE International Conference on Artificial Intelligence and eXtended and Virtual Reality (AIxVR).

[52] Arrieta A.B., Díaz-Rodríguez N., Del Ser J., Bennetot A., Tabik S., Barbado A., García S., Gil-López S., Molina D., Benjamins R. (2020). Explainable Artificial Intelligence (XAI): Concepts, taxonomies, opportunities and challenges toward responsible AI. Inf. Fusion.

[53] Harari R.E., Altaweel A., Ahram T., Keehner M., Shokoohi H. (2025). A randomized controlled trial on evaluating clinician-supervised generative AI for decision support. Int. J. Med. Inform..

[54] Harari R.E., Schulwolf S.L., Borges P., Salmani H., Hosseini F., Bailey S.K.T., Quach B., Nohelty E., Park S., Verma Y. (2025). Applications of Augmented Reality for Prehospital Emergency Care: Systematic Review of Randomized Controlled Trials. JMIR XR Spat. Comput..

[55] Harari R.E., Altaweel A., Anderson E., Pozner C., Grossmann R., Goldsmith A., Shokoohi H. (2025). Augmented Reality in Enhancing Operating Room Crisis Checklist Adherence: Randomized Comparative Efficacy Study. JMIR XR Spat. Comput..

[56] Harari R.E., Kian C., Ebnali-Heidari M., Mazloumi A. User experience in immersive VR-based serious game: An application in highly automated driving training. In Proceedings of International Conference on Applied Human Factors and Ergonomics.

[57] Harari R.E., Paladugu P., Miccile C., Park S.H., Burian B., Yule S., Dias R.D. (2023). Extended reality applications for space health. Aerosp. Med. Hum. Perform..

[58] Harari R.E., Dias R.D., Kennedy-Metz L.R., Varni G., Gombolay M., Yule S., Salas E., Zenati M.A. (2024). Deep learning analysis of surgical video recordings to assess nontechnical skills. JAMA Netw. Open.

[59] Harari R., Smith S.E., Bahouth S.M., Lo L., Sury M., Yin M., Schaefer L.F., Wells W., Collins J., Duryea J. (2025). Predicting Knee Osteoarthritis Progression Using Explainable Machine Learning and Clinical Imaging Data. SSRN.

[60] Jafari Z., Harari R.E., Hole G., Kolb B.E., Mohajerani M.H. (2025). Machine Learning Models Can Predict Tinnitus and Noise-Induced Hearing Loss. Ear Hear..

